# Response to letter to the editor

**DOI:** 10.1186/s13012-021-01148-6

**Published:** 2021-08-16

**Authors:** Ann Dadich, Annika Piper, Dominiek Coates

**Affiliations:** 1 School of Business, Western Sydney University, Locked Bag 1797, Penrith, NSW 2751 Australia; 2 School of Nursing and Midwifery, University of Technology Sydney, PO Box 123, Broadway, NSW 2007 Australia

Dear Editor,

Thank you for the opportunity to respond to the letter penned by Dr Blankstein Breman and colleagues, which highlighted issues regarding our scoping review on implementation science in maternity care. Similarly, we wish to thank Dr Blankstein Breman and colleagues for their interest in our contribution to Implementation Science.

Our scoping review ‘appraise[d] the scientific study of methods to promote the systematic uptake of evidence-based interventions in maternity care by clarifying if and how implementation science theories, models, and frameworks are used’ [1]. Akin to other scoping reviews [2], we used Nilsen’s [3] categories to consider whether and how publications on implementation science in maternity care used a theory, model, and/or framework to guide implementation. These categories include classic theories, determinant frameworks, implementation theories, evaluation frameworks, and process models. Of the 1181 publications identified, 158 were included in our review.

Dr Blankstein Breman and colleagues expressed concern with our ‘study’s methodology’ [4]. They indicated that it failed to identify ‘important implementation research studies on critical interventions for the reduction of maternal morbidity and mortality globally… [and] published studies utilizing implementation research frameworks and theories in maternity care’. As such, they recommended the use of ‘broader search terms’ to include a number of ‘important implementation research studies’ they were familiar with.

Although we appreciate this point and the suggested publications, our scoping review purposely focused on publications pertaining to ‘maternity’ care for four key reasons. First, this term is part of international vernacular, offering the capacity to detect publications conducted by midwives (e.g., in the United Kingdom and Australia) as well as obstetric nurses (e.g., in the United States). Second, and as justified in our article, a relatively more inclusive approach proved to unhelpfully dilute the relevance of the publications that were identified. Third, as a scoping (rather than a systematic) review, our aim was not to identify every relevant publication, but rather, to analyse a selection of publications—this limitation was duly noted in our article. As Dr Blankstein Breman and colleagues attested, ‘The breadth of maternity care settings, and diversity of implementation constraints between settings, makes it challenging to map this literature in one review paper’ [4]—this comment supports our approach. And fourth, this scoping review involved screening 1181 publications, of which 158 were included—this represents a substantial corpus of literature to helpfully map the use of implementation science in maternity care and base our conclusions, particularly given the expansive scope that they collectively represented.

Despite their methodological concern, Dr Blankstein Breman and colleagues did *not* fault our findings. They agreed that ‘maternity care is in great need of implementation research to close… gaps’. Furthermore, they indicated that we ‘*rightly* note[d] the need to promote the consistent application of implementation science theories and frameworks’ (emphasis added). Given their familiarity with, and knowledge of the articles they helpfully suggested, the take-home-message appears to be the same. As Dr Blankstein Breman and colleagues noted, our study concluded that ‘there remains much work to be done to support implementation in maternity care’. We believe that, had we included the publications they kindly suggested, the key findings from our article would *not* have changed. As such, we argue that our study culminated with robust findings that withstand this critique.

Like all good research, our article was intended to promote discussion and debate on implementation science in maternity care. We thank Dr Blankstein Breman and colleagues for engaging in this discussion and their commitment to implementation science in maternity care. We encourage the Implementation Science community to similarly advance this discipline in the important field of maternity care, for the reasons cited in our article.

Sincerely,

A/Prof. Ann Dadich, Mrs Annika Piper, and Adjunct A/Prof. Dominiek Coates

## Data Availability

Not applicable.
